# Mental Health in Pre-Adolescents with Cerebral Palsy: Exploring the Strengths and Difficulties Questionnaire as a Screening Tool in a Follow-Up Study including Multi-Informants

**DOI:** 10.3390/children9071009

**Published:** 2022-07-06

**Authors:** Hanne Marit Bjorgaas, Irene Bircow Elgen, Mari Hysing

**Affiliations:** 1 Department of Pediatric Neurology, Habu Stavanger, Stavanger University Hospital, Stavanger HF, 4068 Stavanger, Norway; 2 Department of Clinical Medicine, Bergen University Hospital, 5020 Bergen, Norway; irene.bircow.elgen@helse-bergen.no; 3 Department of Psychosocial Science, University of Bergen, 5020 Bergen, Norway; mari.hysing@uib.no

**Keywords:** cerebral palsy, mental health, psychiatric disorders, emotional problems, behavioral problems, trajectory, multi-informants

## Abstract

There is a high prevalence of mental health problems in children with Cerebral Palsy (CP). Still, knowledge regarding the trajectory of mental health problems throughout childhood and differences according to informants is lacking. There is also a need for more knowledge regarding the validity of mental health screening tools. In the present study, we assessed changes in parent-rated mental health problems in a cohort of 36 children with CP from school-starting age to pre-adolescence and differences in mental health problems according to informants. Further, we assessed the validity of the Strengths and Difficulties Questionnaire (SDQ) for psychiatric disorders. The study cohort was assessed using the SDQ and a child psychiatric diagnostic instrument at school-starting age and at pre-adolescence. Mean parental SDQ scores increased significantly for emotional, hyperactivity and total problems. Self-reported impact of mental health problems was significantly lower than parent-reported impact, and parents and pre-adolescents reported significantly higher mean scores than teachers for emotional problems, conduct problems and total problem scores. Validated against psychiatric disorders, the SDQ was satisfactory for screening children with CP for risk of psychiatric disorders at pre-adolescence. We recommend that mental health screening be integrated into the regular follow-up for children with CP.

## 1. Introduction

There is an increasing awareness of co-existing mental health problems in children and adolescents with Cerebral Palsy (CP) [1,2,3], which is one of the most common motor disorders in childhood [4,5]. In fact, a review study reported a pooled prevalence of mental health problems in one in three children with CP [6]. The prevalence remained high throughout childhood and even into adulthood [7], with a much higher prevalence of depression, anxiety and behavioral problems in adults with CP than in those without CP [8]. In a Danish population study of 446 children with CP aged 8–15 years using the Child Behavior Checklist (CBCL), almost half were categorized as high scorers for mental health problems as opposed to 15% of the general childhood population [9]. Further, an increase in the prevalence of psychiatric disorders from 57% to 79%, with a shift towards an increase in emotional disorders, was reported when assessing the current cohort of children with CP at school-starting age and again at pre-adolescence [10,11].

Screening tools, such as the Strengths and Difficulties Questionnaire (SDQ) [12,13], have been used to assess mental health in cohorts of children and adolescents with CP throughout childhood [1,14]. SDQ is a brief screening questionnaire for mental health problems, including versions for self-report, parent-report and teacher-report. Differences in mental health problems according to informants have been reported, such as in a study of youth with CP, wherein mothers reported more severe mental health problems than the young person himself/herself [15]. Given the high prevalence of mental health problems in children with CP, mental health screening has been suggested [9], in which case validated screening tools would be needed. We have previously performed a validation of the SDQ in children with CP at school-starting age, finding satisfactory sensitivity and low specificity [16]. However, knowledge regarding the validity of mental health screening tools for children with CP later in childhood is lacking.

In this study, we want to assess the validity of SDQ as a screening tool in children with CP at pre-adolescence. Firstly, to assess changes in parent-rated mental health problems from school-starting age to pre-adolescence in a cohort of children with CP; secondly, to assess differences in mental health problems across informants at pre-adolescence; and thirdly, to investigate the validity of the SDQ in comparison to psychiatric disorders assessed using a child-psychiatric diagnostic instrument at pre-adolescence.

## 2. Materials and Methods

### 2.1. Population

A cohort of 67 out of 98 invited children with CP, born between 2001 and 2003 and living in the Western Health Region of Norway, was assessed for mental health at school-starting age [10]. In this cohort, 56 children with GMFCS levels I–IV were invited for assessment again at pre-adolescence [11]. The prevalence of child psychiatric disorders was assessed using a semi-structured diagnostic interview with parents as informants at school-starting age and again at pre-adolescence. Likewise, mental health was assessed in the cohort using parental SDQ at school-starting age and at pre-adolescence, as well as SDQ self-report and teacher-report at pre-adolescence. In the present study, we included children who took part in the assessment using a child psychiatric diagnostic instrument at school-starting age and at pre-adolescence as well as parental SDQ at both school-starting age and at pre-adolescence, and SDQ self- and teacher- reports at pre-adolescence.

### 2.2. Classification, Functional Levels and Medical Information

Cerebral Palsy was classified according to ICD-10 criteria G80.0–G80.9 with the following subgroups: spastic bilateral and unilateral, dyskinetic, atactic or not further classified. Information regarding type and severity of CP, co-existing conditions and demographic details was collected at school starting age, and any changes were recorded at pre-adolescence.

Classification of gross motor function was based on self-initiated movement, functional limitations and the use of mobility devices in everyday life, using the Gross Motor Function Classification System (GMFCS), which distinguishes five groups [17]. In the present study, classification was grouped as follows: light disability (GMFCS levels I and II), moderate disability (GMFCS levels III and IV) and severe disability (GMFCS level V). Children with light disability would be able to walk but would need some support while negotiating stairs. Moderate disability would involve reliance on a walking aid or wheelchair. We recorded functional classification given in the medical record. If classification was not available prior to the study, this was carried out during the medical examination [10]. Information regarding Intellectual Disability (ID) and communication difficulties was recorded through the medical record, and parents verified this information during the interview.

### 2.3. Mental Health Screening at Ages Seven and Eleven

The Strengths and Difficulties Questionnaire (SDQ) [18], is a brief screening questionnaire for mental health problems, consisting of 25 items. Four of the items record problem domains, each including five items, and one pro-social domain, including five items. Each item can be answered with “not true”, “somewhat true”, or “certainly true” rated 0–2 for negatively worded items, and inversely 2–0 for positively worded items. The problem domains are: Hyperactivity problems, including items such as inattentiveness and distractibility; Conduct problems, including items such as disobedience and temper tantrums; Emotional problems, including items such as anxiety and worry; and Peer problems, including items such as loneliness and preferring adult company. Prosocial behavior consists of items such as being helpful and kind. Combining the four problem subscales (0–10) computes the total difficulties score (TDS) (0–40), and higher mean scores indicate more mental health problems. The SDQ also includes an impact score (IS) which measures the impact of mental health problems on the child’s functioning in daily life. In the present study, parents filled in the SDQ at school-starting age and at pre-adolescence. Likewise, teachers and the children themselves filled in the SDQ at pre-adolescence.

A Norwegian childhood population was used as a reference group for the present study, as Norwegian standard cut-off scores are not yet available for SDQ [19]. A score at or above the 90th percentile of the reference group was defined as screen-positive and compared to psychiatric disorders assessed according to DSM-IV criteria for each of the problem score subscales. Parental reports were available at school-starting age and at pre-adolescence, and these were used for assessing the trajectory of mental health problems across the study period.

### 2.4. Psychiatric Disorders Assessed at Pre-Adolescence

The SDQ was validated against psychiatric disorders assessed using the Schedule for Affective Disorders and Schizophrenia for School-Age Children: Present and Lifetime Version (6–18) 10.04.00 (Kiddie-SADS) [20]. This is a semi-structured child psychiatric diagnostic interview designed to unveil psychiatric symptoms within all categories of child psychiatric disorders. We drew conclusions according to the Diagnostic and Statistical Manual of Mental Disorders, Fourth Edition (DSM-IV), as a Norwegian version of the Kiddie-SADS using DSM-V criteria was not available at the assessment time. Parents of all children in the cohort were interviewed according to the Kiddie-SADS, and a psychiatric disorder was ascertained if criteria listed in DSM-IV for each specific diagnosis were fulfilled, including severity and duration of specific symptoms.

### 2.5. Statistical Analysis

We used descriptive analyses to describe the study population, and *t*-tests to compare SDQ means of the study population between school-starting age and pre-adolescence. Likewise, *t*-tests were used to compare SDQ means recorded by the child themself, the parents and teachers at pre-adolescence. Mental health problems recorded using the SDQ were compared to psychiatric disorders (DSM-IV criteria) for the following symptom–disorder pairs: SDQ total problem scores against any psychiatric disorder, SDQ total problem scores against any behavioral disorder, SDQ total problem scores against any emotional disorder and SDQ emotional problem scores against any emotional disorder. For validation of the SDQ as a screening instrument for children with CP at pre-adolescence, we used the 90th percentile for the reference group as cut-off scores. Using cross-tabulation, SDQ values above the 90th percentile were assessed against the dichotomized prevalence of any psychiatric disorder, any emotional disorder and any behavioral disorder. Sensitivity was defined as the proportion of actual positives that were correctly identified as such; Specificity was defined as the proportion of actual negatives that were correctly identified as such. Positive Predictive Value (PPV) was defined as the proportion of positive tests that were true positives; Negative Predictive Value was defined as the proportion of negative tests that were true negatives. Values at or above 0.8 were considered good [21]. The SPSS version 24 was used for statistical analysis.

## 3. Results

The study cohort was derived from an original cohort of 67 children with CP including all GMFCS levels. The children were born between 2001 and 2003 and were living in the Western Health Region of Norway, where an assessment of mental health had been made at school-starting age. In the present study, 47 children with GMFCS levels I–IV who had undergone assessment of psychiatric disorders at school-starting age and at pre-adolescence and who had been assessed using the SDQ mental health questionnaire at school-starting age were invited. The present study included 36 children (77%) for whom a complete dataset was available, including assessment for psychiatric disorders using a diagnostic instrument at school-starting age and at pre-adolescence, parental SDQs both at school starting age and pre-adolescence, as well as self- and teacher reports at pre-adolescence. The majority of children in the study cohort were boys, and three in four children had a light motor disability (Table 1).

In the group of children who were omitted from the present study due to incomplete datasets, there were more girls and fewer children had ID, GMFCS levels III–IV and communication problems. In the present study, parents reported significant increases in mean SDQ scores between school-starting age and pre-adolescence for emotional, hyperactivity and total problems, whereas impact scores remained the same across the study period (Table 2).

Self- and parent-reported scores were significantly higher than teacher-reported scores for emotional, conduct and total problems (Table 3).

Further, parents reported significantly higher impact scores than the children themselves, and parental impact scores were significantly higher than those reported by the teachers (Table 3). Validated against psychiatric disorders at pre-adolescence, Specificity and Positive Predictive Value (PPV) were satisfactory for SDQ total problem scores (Table 4). Likewise, PPV and Specificity was satisfactory for SDQ total problem scores assessed against behavioral disorders, whereas SDQ total problem scores assessed against emotional disorders yielded satisfactory results for Sensitivity. For SDQ emotional problems assessed against emotional disorders, PPV and Specificity were satisfactory (Table 4).

## 4. Discussion

In the present study of children with CP, parents reported significantly increased scores for emotional problems, hyperactivity problems and total problems across childhood. They also reported significantly higher mental health impact scores than the children themselves and the teachers. The validity of the SDQ total problem score was satisfactory for assessing mental health in children with CP at pre-adolescence.

The rate of mental health problems increased across childhood in the present study, with significantly higher rates for emotional and total problems scores at pre-adolescence compared to school-starting age [16]. Similar findings were reported in a longitudinal study including 6–18-year-olds using the SDQ, in which the authors reported a 7% transition from normal to abnormal on the emotional scale [22]. In a recent paper assessing mental health in 6–17 year old children with CP [23], odds for anxiety and behavioral problems remained increased compared to controls. For other neurological conditions, too, such as epilepsy, a high prevalence of emotional disorders has been reported, associated with high levels of parental stress [24]. In fact, a recent study in children with neurodevelopmental disorders, undertaken during the COVID-19 pandemic, found an increase in internalizing and externalizing symptoms as well as increased parental stress compared to the pre-COVID-19 situation [25]. In the present study, informant differences were found for emotional problems, with more emotional problems reported by the parents and the children themselves compared to the teachers. This is similar to a previous study reporting low to moderate agreement among informants on internalizing problems [26]. Further, parents reported significantly higher impacts of mental health problems than did the pre-adolescents themselves. Interestingly, a previous study including otherwise normally developing children found that parents reported higher impacts than their children, even if the children reported more mental health symptoms [27]. Differences between parent- and self-reports were also found in a recent study assessing the perception of the CP condition [28], in which children reported significantly less perceived impact of the condition than their parents. The children have never experienced a life without CP, which perhaps could explain some of the differences reported between parents and the children themselves. In fact, another study found that agreement between parent- and self-reported mental health problems was associated with the child living with both parents, having good communication between child and parents, as well as having parents who were engaged in the child’s activities [27]. In the present study, parents and children reported more mental health problems than teachers, and our findings could indicate that mental health problems could be more prominent at home than in school. The fact that home and school are different settings for observing children [29] could explain some of the discrepancies, with teachers scoring lower than parents and children themselves. School settings are often more structured than home settings, and children with CP are often entitled to support from an assistant teacher in school and not at home. Another aspect could be that teachers tend to attribute symptoms of maladaptive behaviors to the CP condition itself, resulting in a “diagnostic overshadowing” of mental health problems. This has been described previously for children with neurodevelopmental disorders and co-existing ADHD [30].

In the present study, SDQ total difficulties score assessed against any psychiatric disorder yielded good Specificity and PPV, whereas Sensitivity was moderate. The use of Sensitivity and Specificity metrics in clinical practices has been discussed, as the prevalence of a target disorder in the population could influence Sensitivity and Specificity [21], arguing that the PPV perhaps would be the most useful value in clinical practice [31]. In a population of children with CP, where we know that the prevalence of psychiatric disorders is high, the PPV may therefore be a better value to emphasize when choosing a screening tool for the assessment of mental health problems in children with CP [32]. 

Given the high and increasing prevalence of mental health problems throughout childhood [11,16,33], awareness of mental health problems in children with CP within both the health and school systems seems warranted. For otherwise normally developing children, early mental health counselling and interventions in schools have proven beneficial, not only for the well-being of the children, but also from an economic point of view [34]. The conclusion therefore seems warranted that early detection of mental health problems could be beneficial for children with CP, in line with a recent Danish study which concluded that screening for mental health problems should be offered to all children with CP [9]. Given the high prevalence of psychiatric disorders in children with CP, screening for mental health problems appears to be relevant at given intervals across childhood. Studies supporting specific age intervals for assessing mental health in children with CP are to our knowledge lacking. However, we suggest mental health screening at school-starting age, as this coincides with the timing for cognitive assessments in preparation for an optimal transition into school. Likewise, mental health screening at pre-adolescence could provide useful information in preparation for the expected demands of adolescence. Acknowledging the complexity of the CP condition, with a number of co-existing medical problems accompanying it [5], a multi-disciplinary service could perhaps be the preferred means of investigating psychiatric disorders in children who have screened above the cut-off for mental health problems. The SDQ is a short screening tool, which is easy to complete for children and young people themselves, as well as for parents and teachers. It could perhaps be used within the framework of existing medical, multi-disciplinary follow-up services as a screening tool to be filled in by parents and by the children themselves when possible. If needed, additional information from teachers could be obtained. The use of multi-informants could prove beneficial, as a high burden of care and mental health challenges have been described in these families, increasing the risk of psychopathology [35,36,37].

This study indicates that the SDQ total problem score has a Positive Predictive Value (PPV) of 100%, indicating that the properties assessed to detect pre-adolescents with CP at risk of developing psychiatric disorders are satisfactory. We suggest that screening for mental health problems should be offered to all children and young people with CP.

## 5. Strengths and Limitations

To our knowledge, the present study is unique in its longitudinal design, including child psychiatric diagnostic assessments. Numbers are, however, small and limit the statistical power. Conclusions should therefore be drawn carefully. The distribution of types of CP and the prevalence of Intellectual Disability (ID) is comparable to previous epidemiological studies [5], however, the cohort is skewed towards light motor disability (GMFCS levels I-II) [5]. The burden of care many parents of severely affected children experience could perhaps explain the drop-out rate of children with GMFCS levels III–IV, which is similar to drop-out rates in other studies involving children with severe conditions [38,39]. In the present study, children with GMFCS levels V were omitted [10], as neither the Kiddie-SADS nor the SDQ would be suitable for this group of children, and conclusions regarding mental health problems in the most severely affected children should be drawn cautiously. Despite the limitations of the study, we have stated that there is a significant increase in mental health problems across childhood and that the SDQ could be a useful screening tool for detecting children with CP at risk of developing one or more psychiatric disorders.

## 6. Conclusions

In the present study, parents reported significant increase in mental health problems in children with CP across childhood. They also reported significantly higher impacts of mental health problems than the children themselves and teachers. We recommend multi-informant screening for mental health problems in children with CP at regular intervals and suggest screening at school-starting age and at pre-adolescence. The SDQ total problems score seems to include satisfactory properties for screening children with CP for the risk of developing psychiatric disorders.

## Figures and Tables

**Table 1:** Descriptive characteristics for the cohort of children with CP assessed for mental health problems at ages seven and eleven.

N = 36	Number (%)	
Boys	22	(61)
ID ^i^	11	(31)
GMFCS ^ii^ levels	28	(78)
GMFCS levels	8	(22)
GMFCS level	0	
Communication problems	18	(50)
**Type of CP**		
Spastic unilateral	12	(33)
Spastic bilateral	20	(56)
Dyskinetic or Atactic	4	(11)
Psychiatric Disorder	26	(72)

^i^ Intellectual Disability. ^ii^ Gross Motor Function Classification System.

**Table 2:** Parent-reported mean scores and mean differences assessed using the Strengths and Difficulties Questionnaire (SDQ) at ages seven and eleven for a cohort of children with Cerebral Palsy.

N = 36				
SDQ Scores	Mean Age 7	Mean Age 11	Mean Diff. ^i^	*p*-Value
Emotional problems	2.35	4	1.65	0.00 ^ii,^*
Conduct problems	1.5	1.5	0	1
Hyperactivity problems	4.42	5.23	0.81	0.05 *
Peer problems	2.63	2.63	0	1
Prosocial behavior	8.17	8.63	0.47	0.13
Total problem score	10.8	13.23	2.43	0.01 *
Impact	2.71	3.52	0.81	0.17

^i^ Mean Difference between ages seven and eleven. ^ii^ Significance level at or below 0.05. * Significance level at or below 0.05.

**Table 3:** Mean scores and mean differences between self, parent and teacher ratings of SDQ at age 11 for children with Cerebral Palsy (n = 36).

	Self Rated	Parent Rated	Teacher Rated	Self-Parent ^i^		Self-Teacher ^ii^		Parent-Teacher ^iii^	
SDQ Problem Scores	Mean (SD)	Mean (SD)	Mean (SD)	Mean Diff.	*p*-Value	Mean diff.	*p*-Value	Mean diff.	*p*-Value
Emotional problems	3.8 (2.6)	3.9 (2.9)	2.1 (2.2)	0.09	0.71	1.54	0.01 *	1.76	0.00 ^iv,^*
Conduct problems	1.9 (1.9)	1.5 (1.2)	0.7 (1.2)	0.44	0.18	1.04	0.02 *	0.79	0.00 *
Hyperactivity problems	4.7 (2.8)	4.8 (2.8)	4.0 (3.1)	0.13	0.75	0.39	0.48	0.79	0.11
Peer problems	2.2 (2.0)	2.8 (2.9)	2.0 (2.6)	0.56	0.07	0.19	0.59	0.35	0.34
Prosocial behavior	8.4 (1.7)	8.6 (2.3)	8.2 (2.2)	0.16	0.48	0.39	0.34	0.59	0.12
Total problem score	12.7 (7.5)	12.3 (7.6)	8.6 (5.9)	0.34	0.66	2.77	0.03 *	3.69	0.03 *
Impact score	2.6 (2.5)	3.4 (2.5)	1.9 (1.6)	0.88	0.04 *	0.75	0.07	1.67	0.00 *

^i^ Mean differences between self- and parent rated SDQ scores. ^ii^ Mean differences between self- and teacher rated SDQ scores. ^iii^ Mean differences between parent- and teacher rated SDQ scores. ^iv^ Significance level at or below 0.05. * Significance level at or below 0.05.

**Table 4:** Validity of parent-reported Strengths and Difficulties Questionnaires (SDQs) using the 90th percentile cut-off score assessed according to Psychiatric Disorders in children with Cerebral Palsy at pre-adolescence.

SDQ vs. Psychiatric Disorder	Sensitivity N (%)	Specificity N (%)	PPV ^i^ N (%)	NPV ^ii^ N (%)
SDQ total problem score vs. any Psychiatric Disorder	18/26 (69)	9/9 (100.0)	18/18 (100)	9/17 (53)
SDQ total problem score vs. any Behavioral Disorder	16/23 (70)	10/12 (83)	16/18 (90)	10/17 (59)
SDQ total problem score vs. any Emotional Disorder	11/13 (85)	15/22 (68)	11/18 (61)	15/17 (88)
SDQ emotional problem score vs. any Emotional Disorder	11/19 (58)	14/16 (88)	11/13 (85)	14/20 (64)

^i^ Positive Predictive Value. ^ii^ Negative Predictive Value.

## Data Availability

The data cannot be made available as they include personal information.
